# Functional vs Structural Cortical Deficit Pattern Biomarkers for Major Depressive Disorder

**DOI:** 10.1001/jamapsychiatry.2025.0192

**Published:** 2025-04-02

**Authors:** Peter Kochunov, Bhim M. Adhikari, David Keator, Daniel Amen, Si Gao, Nicole R. Karcher, Demetrio Labate, Robert Azencott, Yewen Huang, Hussain Syed, Hongjie Ke, Paul M. Thompson, Danny J. J. Wang, Braxton D. Mitchell, Jessica A. Turner, Theo G.M. van Erp, Neda Jahanshad, Yizhou Ma, Xiaoming Du, William Burroughs, Shuo Chen, Tianzhou Ma, Jair C. Soares, L. Elliot Hong

**Affiliations:** 1 Department of Psychiatry and Behavioral Sciences, UTHealth Houston School of Behavioral Health Sciences, University of Texas Health Science Center at Houston, Houston; 2 Amen Clinics Inc, Costa Mesa, California; 3 Department of Psychiatry and Human Behavior, University of California, Irvine,; 4 Change Your Brain Change Your Life Foundation, Costa Mesa, California; 5 Department of Psychiatry, Washington University in St Louis School of Medicine, St Louis, Missouri; 6 Departments of Mathematics, University of Houston, Houston, Texas; 7 Department of Biostatistics, University of Maryland College Park, College Park; 8 Imaging Genetics Center, Mark and Mary Stevens Neuroimaging and Informatics Institute, Keck School of Medicine, University of Southern California, Marina del Rey; 9 Laboratory of Functional MRI Technology, Mark and Mary Stevens Neuroimaging and Informatics Institute, Keck School of Medicine, University of Southern California, Los Angeles; 10 Department of Medicine, University of Maryland School of Medicine, Baltimore; 11 Geriatrics Research and Education Clinical Center, Baltimore Veterans Administration Medical Center, Baltimore, Maryland; 12 Department of Psychiatry and Behavioral Science, The Ohio State University College of Medicine, Columbus; 13 Clinical Translational Neuroscience Laboratory, Department of Psychiatry and Human Behavior, University of California, Irvine; 14 Center for the Neurobiology of Learning and Memory, University of California Irvine; 15 Department of Psychiatry, University of Maryland School of Medicine, Baltimore

## Abstract

**Importance:**

Major depressive disorder (MDD) is a severe mental illness characterized more by functional rather than structural brain abnormalities. The pattern of regional homogeneity (ReHo) deficits in MDD may relate to underlying regional hypoperfusion. Capturing this functional deficit pattern provides a brain pattern–based biomarker for MDD that is linked to the underlying pathophysiology.

**Objective:**

To examine whether cortical ReHo patterns provide a replicable biomarker for MDD that is more sensitive than reduced cortical thickness and evaluate whether the ReHo MDD deficit pattern reflects regional cerebral blood flow (RCBF) deficit patterns in MDD and whether a regional vulnerability index (RVI) thus constructed may provide a concise brain pattern-based biomarker for MDD.

**Design, Settings, and Participants:**

The UK Biobank (UKBB) participants had ReHo and structural measurements. Participants from the Enhancing Neuroimaging Genetics Through Meta-Analysis (ENIGMA) Consortium were included for measuring the MDD structural cortical deficit pattern. The UKBB ReHo and ENIGMA cortical thickness effect sizes for MDD were used to test the deficit patterns in the Amish Connectome Project (ACP) with ReHo, structural, and RCBF data. Finally, the Ament Clinic Inc (ACI) sample had RCBF data measured using single-photon emission computed tomography. Data were analyzed from August 2021 to September 2024.

**Exposures:**

ReHo and structural measurements.

**Results:**

Included in this analysis were 4 datasets: (1) UKBB (N = 4810 participants; 2220 with recurrent MDD and 2590 controls; mean [SD] age, 63.0 [7.5] years; 1121 female [50%]), (2) ENIGMA (N = 10 115 participants; 2148 with MDD and 7957 healthy controls; mean [SD] age, 39.9 [10.0] years; 5927 female [59%]), (3) ACP (N = 204 participants; 68 with a lifetime diagnosis of MDD and 136 controls; mean [SD] age, 41.0 [14.5] years; 104 female [51%]), and (4) ACI (N = 372 participants; 296 with recurrent MDD and 76 controls; mean [SD] age, 45.3 [17.2] years; 189 female [51%]). MDD participants had lower cortical ReHo in the cingulum, superior temporal lobe, frontal lobe, and several other areas, with no significant differences in cortical thickness. The regional pattern of ReHo MDD effect sizes was significantly correlated with that of RCBF obtained from 2 independent datasets (Pearson *r* = 0.52 and Pearson *r *= 0.46; *P* < 10^−4^). ReHo and RCBF functional RVIs showed numerically stronger effect sizes (Cohen *d* = 0.33-0.90) compared with structural RVIs (Cohen *d* = 0.09-0.20). Elevated ReHo-based RVI-MDD values in individuals with MDD were associated with higher depression symptom severity across cohorts.

**Conclusions and Relevance:**

Results of this case-control study suggest that the ReHo MDD deficit pattern reflected cortical hypoperfusion and was regionally specific in MDD. ReHo-based RVI may serve as a sensitive functional biomarker for MDD.

## Key Points

**Question:**

Does the regional homogeneity (ReHo)–magnetic resonance imaging (MRI) cortical deficit pattern provide a more robust biomarker for major depressive disorder (MDD) than its structural counterpart and inform the underlying, regionally specific lower cerebral blood flow in this disorder?

**Findings:**

In this case-control study including a total of 15 501 participants from 4 datasets, the ReHo/regional cerebral blood flow (RCBF) deficits in individuals with MDD had effect sizes that were 2 to 3 times stronger than those with deficits in regional cortical thickness. The effect sizes for functional regional vulnerability index (RVI) were larger than the effect sizes for any individual regions measured in independent datasets, and the RVI-MDD calculated for ReHo and RCBF data showed similar effect sizes that were stronger than the MDD structural RVI.

**Meaning:**

Results suggest a highly reproducible regionally specific hypoperfusion in MDD, which is likely associated with ReHo deficits in people with MDD; the ReHo-based RVI may serve as a novel biomarker to study MDD.

## Teaser

This case-control study investigates if a regional homogeneity–magnetic resonance imaging cortical deficit pattern provides a more robust biomarker for major depressive disorder (MDD) than its structural counterpart and informs the underlying, regionally specific lower cerebral blood flow in this disorder.

## Introduction

Major depressive disorder (MDD) is the most common severe mental illness worldwide, with a lifetime prevalence of 10% to 30%.^1^ Despite this high incidence, the neuroimaging findings in MDD were historically heterogeneous and had poor reproducibility.^2^ MDD does not exert a strong neurodegenerative effect on brain structure, nor is there a strong interaction with common imaging biomarkers of brain aging.^3^ Large-scale meta-analyses from the Enhancing Neuro Imaging Genetics Through Meta Analyses (ENIGMA) suggest that the structural effects of MDD on the brain are small (Cohen *d* = 0.01-0.14).^4,5,6,7,8,9,10,11^ Instead, MDD-related effects on the brain are likely manifested as reductions in regional cerebral blood flow (RCBF)^12^ especially in cingulate, prefrontal, and temporal areas.^13,14^ As the resting-state functional magnetic resonance imaging (MRI) derived regional homogeneity (ReHo)^15,16^ was linked to RCBF in healthy people,^17^ we hypothesized that the effect sizes (ESs) for cortical ReHo values in participants with MDD may serve as a proxy for RCBF deficit in MDD, which should be more robustly associated with MDD than those observed for structural MRI. We further propose that the reproducibility of ReHo deficit patterns can be used as a basis for constructing novel functional similarity-based biomarkers that measure individual agreements with the expected MDD illness pattern.

**Figure 1.**
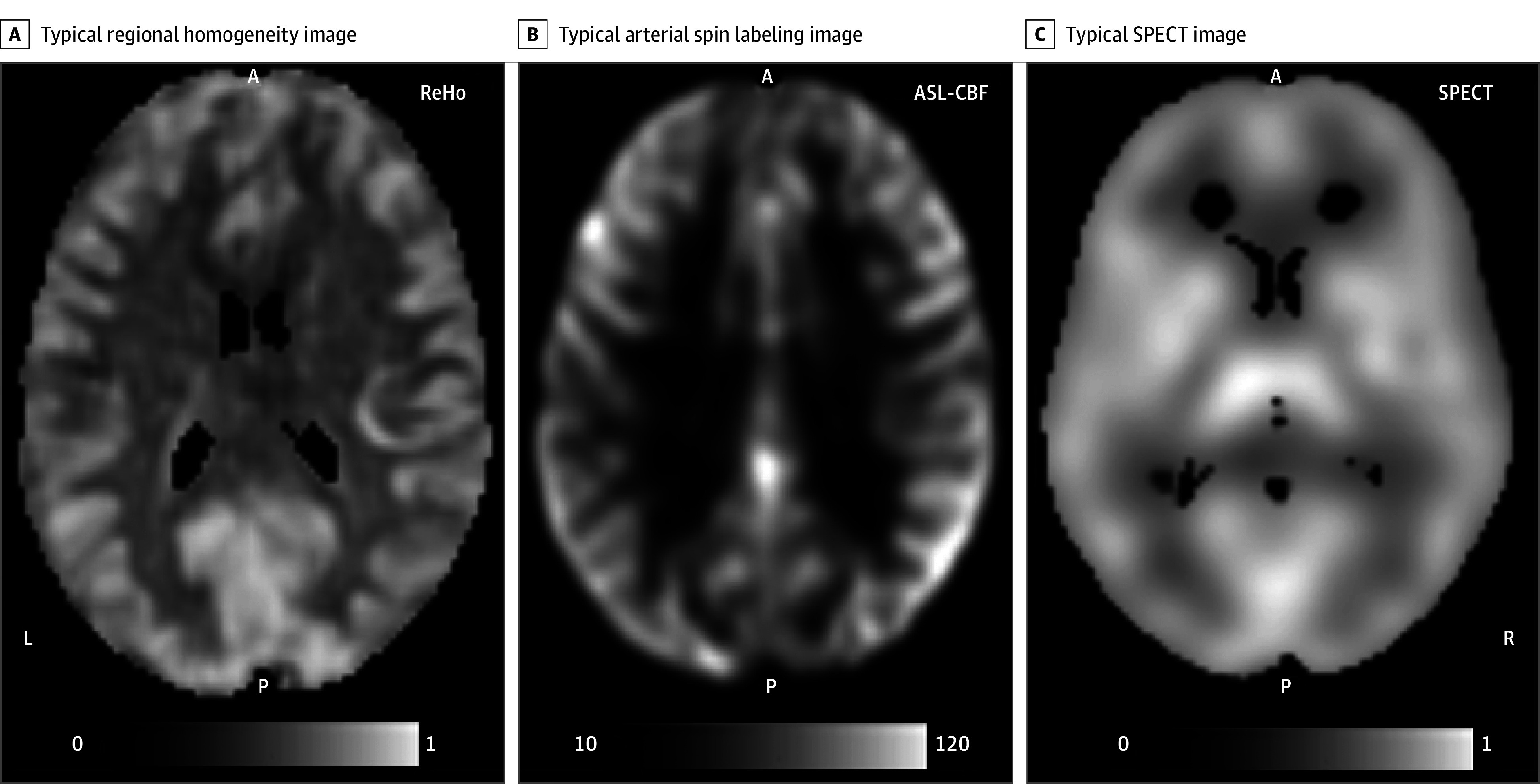
Participant Representative Maps Typical regional homogeneity (ReHo), arterial spin labeling (ASL), and single-photon emission computed tomography (SPECT) single-participant images. Despite entirely different acquisition techniques, resolutions, and scales, there are appreciable similarities between the gross ReHo signal distribution pattern and the ASL-derived cerebral blood flow (CBF) and SPECT-derived CBF patterns.

ReHo quantifies temporal coherence of the resting-state blood-oxygen-level-dependent (BOLD) signal in neighboring voxels from functional MRI data.^18,19^ It has been linked to symptoms and clinical features in MDD and other mental illnesses^20,21,22,23,24^ where lower ReHo in individuals with MDD vs controls were commonly interpreted as deficits in local neural activity/connectivity.^21,25,26,27,28^ Whether the magnitude of temporal correlations of BOLD signal can be interpreted as deficits in neural connectivity remains speculative.^29,30^ Meanwhile, ReHo findings are robust and replicable and have been validated in human and animal research.^31,32,33,34^ For example, in animal models of MDD, ReHo deficits were reversed or attenuated by administrations of antidepressant medications.^31,32^ Although these findings were interpreted as restoration of normal connectivity levels, there is a lack of direct evidence linking ReHo to local connectivity. Instead, ReHo maps look remarkably like cerebral perfusion (Figure 1). We and others have shown that ReHo measurements are physiologically linked to RCBF and capture 40% to 60% of the variance in underlying RCBF.^17^ RCBF is very sensitive to effects of MDD, and it may be the primary driver of the ReHo changes reported in MDD and other illnesses.^35,36,37,38^ Here, we hypothesized that lower regional ReHo in MDD may capture the regional hypoperfusion in affected individuals. We used several independent samples to evaluate the association of MDD with ReHo and compared the regional MDD-related effect sizes on ReHo to differences in cortical thickness and RCBF observed in MDD.

## Methods

### Study Participants

The testing of the proposed hypotheses required large and inclusive samples of individuals with MDD and controls where ReHo, cortical thickness, and RCBF measures are available for all individuals. However, to the best of our effort, we could not identify 2 such samples. Instead, we used 4 large samples where 1, 2, or 3 measures were available. The UK Biobank (UKBB) sample had ReHo and cortical thickness measures. ENIGMA, largest structural study to date,^8^ provided the cortical thickness MDD effect sizes for measuring the MDD structural deficit pattern. We, therefore, used UKBB ReHo and ENIGMA cortical thickness effect sizes for MDD to test the hypotheses in Amish Connectome Project (ACP)—where ReHo, structural, and RCBF by 3-dimensional arterial spin labeling (3D-ASL) data are available in MDD and controls, and in Amen Clinics Inc (ACI) where RCBF was measured using single-photon emission computed tomography (SPECT) modality. ACP participants provided written informed consent on forms, approved by the institutional review board of University of Maryland Baltimore. ACI participants provided informed consent for their anonymous data to be used in future research, during their initial visit to the clinic. We detailed the demographics, inclusion/exclusion criteria, symptom scale ratings and the sample characteristics in the eMethods in Supplement 1. Regarding participant race and ethnicity, both the UKBB and ACP cohorts were majority White race, and the racial composition for the ACI cohort was not recorded. Therefore, race and ethnicity data were not included in this study.

### Data Acquisition, Processing, and ReHo Analyses in the UKBB and ACP Cohorts

UKBB resting-state functional MRI data were acquired on a 3-T Siemens Skyra scanner with repetition time (TR) of 735 milliseconds, 2.4-mm isotropic voxels, and multiband acceleration factor of 8. ACP participants underwent a similar resting-state functional MRI data acquisition using a 3-T Siemens Prisma scanner with TR of 780 milliseconds, 2-mm isotropic voxels, and multiband acceleration factor of 8 (full protocols in the eMethods in Supplement 1).

The resting-state analysis workflow developed by the ENIGMA consortium was used to process the resting-state functional MRI data; processing steps have been described in full detail in prior publications^39,40^ and the eMethods in Supplement 1. The processed data was then used for ReHo calculations. ReHo was designed to investigate changes in regional temporal coherence in BOLD time-series by calculating voxelwise value, called Kendall coefficients of concordance (KCC).^19^ Considering 27 nearest voxel time series, voxelwise KCC values and hence participant-wise ReHo map was computed in 3-D volumetric space using the AFNI-command 3dReHo. ReHo signals were extracted using cortical regions based on the Desikan-Killiany (DK) atlas. We had no a priori hypotheses with respect to lateralization of the effect of MDD on structural, ReHo, or RCBF measures, and given the high correlation between left-right effect sizes for structural and ReHo measurements (Pearson *r *>0.5), we averaged the left and right value before analyses (eMethods in Supplement 1).

### ASL Data Acquisition, Processing, and CBF Extraction in ACP

The ASL data were acquired using 3-D pseudocontinuous ASL with background-suppressed gradient and spin-echo sequence, consisted of 13 pairs of labeled and control scans (approximate time, 10 minutes) (eMethods in Supplement 1). A 3-D T1-weighted image for anatomical reference and a volume of M0 image without background suppression were also acquired. CBF perfusion was estimated by using a standard single-compartment ASL model, partial volume effects correction was performed with a spatially regularized method^41^; spatial regularization, motion correction, and partial volume corrections were performed in FMRIB Software Library, version 6.0.1 (University of Oxford). Partial volume-corrected CBF maps were used to extract the voxelwise CBF values using DK atlas (eMethods in Supplement 1).

### SPECT Data Acquisition, Processing, and Analyses in ACI

SPECT scans were acquired using Picker Prism XP 3000 (Philips) triple-headed gamma camera with low-energy high-resolution fan beam collimators. For each procedure, a weight-appropriate dose of 99mTc–hexamethylpropyleneamine oxime was administered intravenously at rest (eyes open), and participants were scanned for approximately 30 minutes after injection (eMethods in Supplement 1).

### Cortical Thickness Data Processing and Analysis in the ENIGMA, UKBB, and ACP Cohorts

Structural T1-weighted MRI brain scans collected by the ENIGMA, UKBB, and ACP cohorts were analyzed using the ENIGMA harmonized analysis and quality-control protocol.^42^

### Statistical Analyses

The overall goal of the analyses was to identify ReHo differences between MDD cases and controls. Analyses were performed in 3 steps: (1) measuring regional effect sizes for ReHo in MDD; (2) comparing regional ReHo effect sizes with their corresponding effect sizes in the structural measurements; and (3) evaluating if the similarity to ReHo patterns in deficits may capture RCBF information. Regional effect sizes for ReHo, ASL RCBF, and SPECT RCBF case-control differences were created using DK atlas, after adjusting for age and sex with the R packages effsize^43^ and psych^44^ (R Project for Statistical Computing). The reported significance of correlation coefficients among effect sizes was estimated using spin permutation test. The regional effect sizes for ReHo and structural measurements were used to create the cortical ReHo and thickness RVI scores based on established methods,^45^ using RVIpkg.^46^ All statistical analyses were performed in RStudio, version 4.1.1^47^ (R Project for Statistical Computing) (eMethods in Supplement 1). All *P* values were 2-sided, and *P* value < .05 was considered statistically significant. Data were analyzed from August 2021 to September 2024.

## Results

### Study Population

Included in this analysis were 4 datasets: (1) the UKBB cohort (N = 4810 participants; 2220 with recurrent MDD and 2590 controls; mean [SD] age, 63.0 [7.5] years; 1121 female [50%]; 1099 male [50%]), (2) ENIGMA (N = 10 115 participants; 2148 with MDD and 7957 healthy controls; mean [SD] age, 39.9 [10.0] years; 5927 female [59%]; 4188 male [41%]), (3) ACP (N = 204 participants; 68 with a lifetime diagnosis of MDD and 136 controls; mean [SD] age, 41.0 [14.5] years; 104 female [51%]; 100 male [49%]), and (4) ACI (N = 372 participants; 296 with recurrent MDD and 76 controls; mean [SD] age, 45.3 [17.2] years; 189 female [51%]; 183 male [49%]).

### Comparing MDD—Control Structural vs ReHo Differences

**Figure 2.**
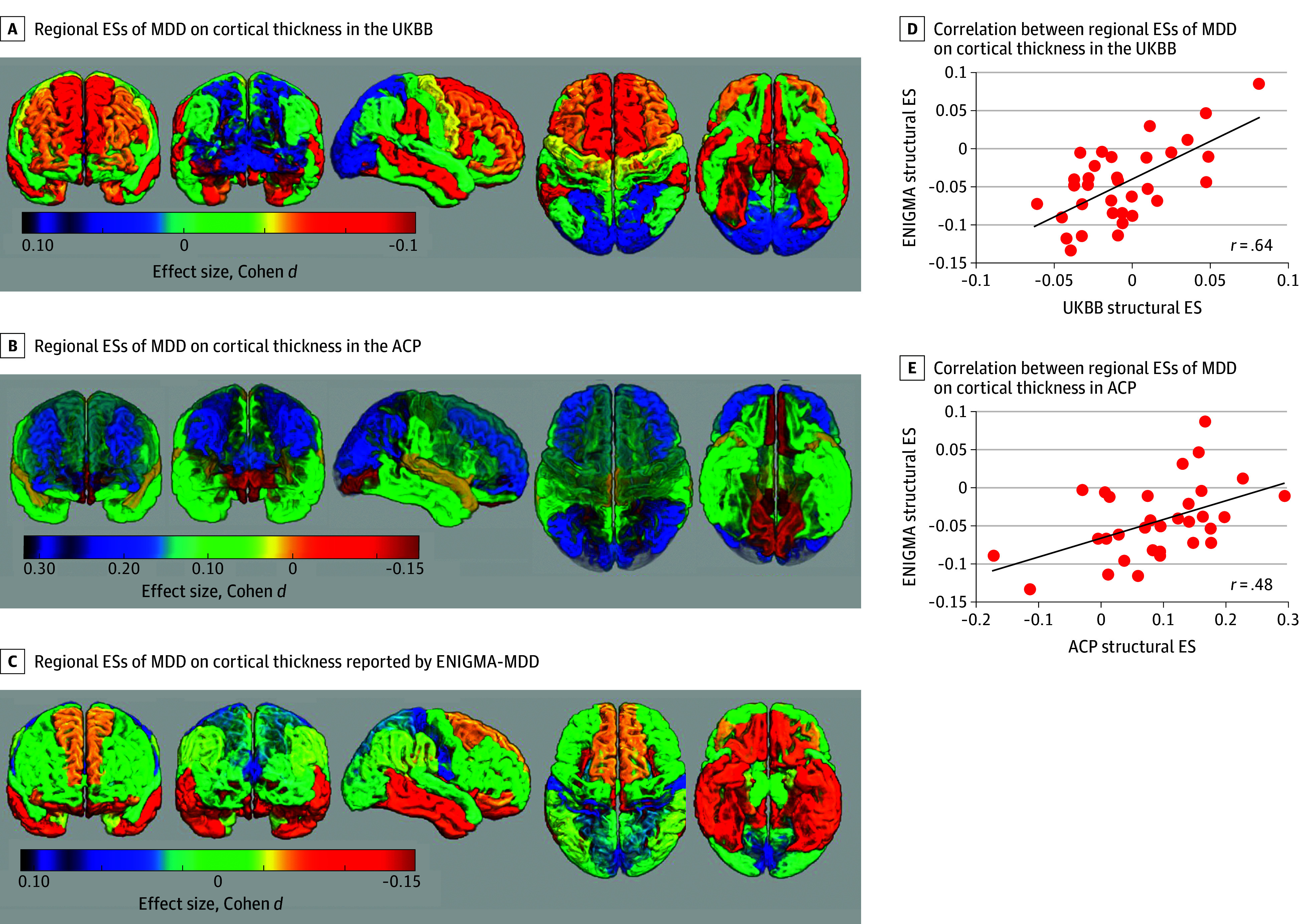
Cortical Thickness Regional Effect Sizes (ESs) Maps Maps of the regional ESs that major depressive disorder (MDD) exerted on cortical thickness in the UK Biobank (UKBB) (A), Amish Connectome Project (ACP) (B), and these reported by the Neuroimaging Genetics Through Meta-Analysis (ENIGMA)–MDD group (C). We observed significant (*P* < .001) correlation between regional ESs of MDD on cortical thickness in the UKBB (D), ACP (E), and these reported by the ENIGMA (right panel D and E).

The pattern of regional effect sizes, represented by Cohen *d*, of MDD on regional cortical thickness reported by ENIGMA was significantly correlated with that observed in UKBB and ACP cohorts (Figure 2), and the correlations of these regional effect sizes were significant (Pearson *r* = 0.64 and Pearson *r* = 0.48, respectively, *P* < .01).

**Figure 3.**
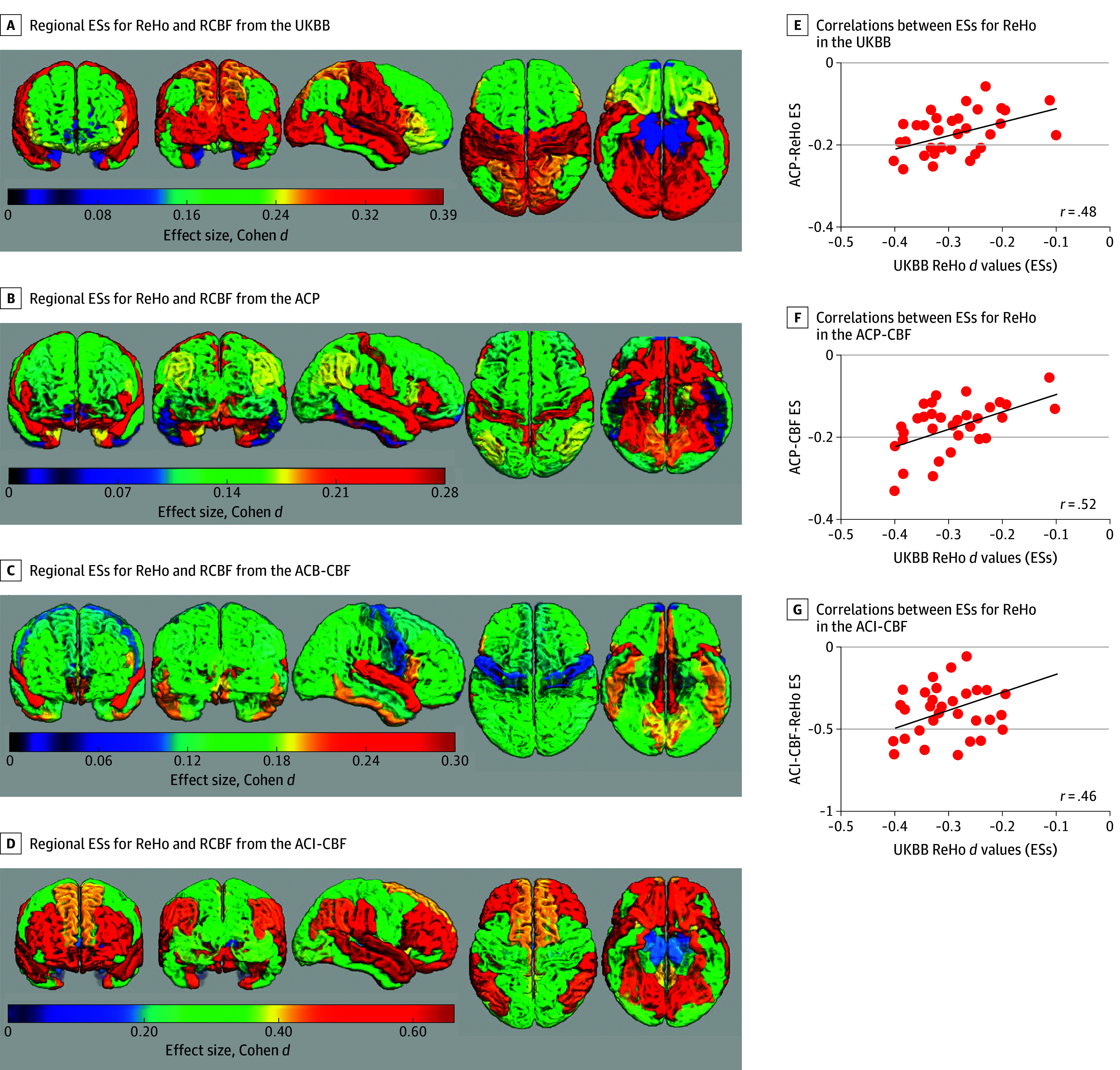
Regional Effect Sizes (ESs) Maps Based on Regional Homogeneity (ReHo) and Cerebral Blood Flow (CBF) Maps of the regional effect sizes (major depressive disorder [MDD]–healthy control [HC]) for ReHo and regional CBF (RCBF) from the UK Biobank (UKBB) (A), Amish Connectome Project (ACP)–ReHo (B), ACP-CBF (C) and ACI-CBF (D) cohorts. We observed significant (all *P* < .001) correlations between regional ESs (MDD-HC) for ReHo in the UKBB and other cohorts (right panel E-G). Note the stronger effect sizes (sign is reversed, A-D) were negative and represented by warmer (eg, red) color, for visualization purposes.

Regional MDD effect sizes for cortical ReHo in the UKBB cohort are shown in Figure 3A and the eTable in Supplement 1. The ReHo values were lower in participants with MDD compared with controls. The mean (SD) effect size for ReHo (Cohen *d* = −0.28 [0.08]) was stronger than that for cortical thickness (0 [0.03]). The largest MDD effect sizes for ReHo were observed for the caudal cingulate, followed by superior and transverse temporal gyri (Cohen *d* = −0.39, *P* =1.4 × 10^−54^; Cohen *d* = −0.39, *P* =3.1 × 10^−53^; and Cohen *d* = −0.38, *P* =2.4 × 10^−51^; respectively).

The ACP participants with MDD had lower ReHo values than controls with the average effect sizes (mean [SD] Cohen *d* = −0.17 [0.02]) (Figure 3B). There were 4 cortical regions with significantly lower ReHo values (caudal anterior cingulate gyrus, insula, posterior cingulate, and precentral gyri) after correction for multiple (N = 33) comparisons (eTable in Supplement 1). The cortical ReHo effect sizes between ACP and UKBB were significantly correlated (Pearson *r* = 0.48; *P* < 5 × 10^−4^) (Figure 3E).

### Comparing MDD—Control ReHo vs RCBF Differences

ACP participants with MDD had lower RCBF values than controls with the average effect sizes across all areas (mean [SD] Cohen *d* = −0.17 [0.06]) (Figure 3C). Two cortical regions with significantly lower RCBF values (anterior and posterior cingulate and insula) were among these with significantly lower ReHo values (eTable in Supplement 1). In ACI (Figure 3D), the average SPECT-based RCBF effect size across all areas (mean [SD] Cohen *d* = −0.37 [0.03]) was stronger than the average effect size observed in ACP 3D-ASL–based RCBF (mean [SD] Cohen *d* = −0.17 [0.06]). The largest effect sizes were observed in superior and middle temporal gyrus (Cohen *d* = −0.66 and Cohen *d *= −0.64), followed by cingulate gyrus (Cohen *d* = −0.58). The regional RCBF effect sizes of MDD from the ACP and ACI were correlated at Pearson *r* = 0.32 (*P* = .01). Here, participants with MDD in the ACI sample were treatment-seeking patients in the specialty mental health clinic, whereas the ACP sample were recruited through a population sampling.

Next, we compared cortical MDD effect size patterns of ReHo vs RCBF by ASL and SPECT (Figure 3A-D). The cortical ReHo effect sizes across regions in UKBB were significantly correlated with both cortical ReHo and RCBF effect sizes in ACP (Pearson *r* = 0.48 and Pearson *r* = 0.52, both *P* < 6 × 10^−3^) (Figure 3E and F) and cortical RCBF effect sizes in ACI (Pearson *r* = 0.46; *P* = 7 × 10^−3^) (Figure 3G). These findings across different samples and imaging methodologies were remarkably similar and the correlation coefficients do not differ significantly across samples (Pearson *r* = 0.48, Pearson *r *= 0.52, and Pearson *r *= 0.57, all *z *score <1.0, all *P* > .5). The RCBF and ReHo ESs in ACP were likewise correlated at Pearson *r* = 0.57; *P* = 3 × 10^−4^) (eTable in Supplement 1). Together, these data provide a strong impression that regional ReHo deficit patterns in MDD substantially reflect the RCBF deficit patterns in MDD. The ACP effect sizes for ReHo and RCBF were significantly correlated with RCBF effect sizes in ACI (ReHo Pearson *r* = 0.32; *P* = .03 and RCBF Pearson *r* = 0.35; *P* = .01).

### Functional RVI for MDD Based on ReHo: Effect Sizes in the ACP and ACI Cohorts

We calculated ReHo-RVI for MDD using effect sizes obtained from UKBB. We calculated structural-RVI for MDD using effect sizes obtained from ENIGMA. ReHo RVI for MDD was significantly elevated in ACP MDD participants compared with controls (Cohen *d* = 0.33; *P* = .007). We also calculated ReHo-RVI in ACI using SPECT RCBF measures. ReHo-based RVI-MDD were significantly higher in patients with MDD compared with controls in ACI (Cohen *d* = 0.90; *P* < 1 × 10^−10^) and in ACP based on ASL (Cohen *d* = 0.36; *P* = .001).

### Comparison of Structural vs Functional RVI

Structural RVI for MDD in each participant from UKBB and ACP was calculated using regional effect sizes for MDD obtained from ENIGMA as the template. Cortical RVI-MDD was significantly elevated in UKBB but not ACP datasets (Cohen *d* = 0.09; *P* = .004 and Cohen *d* = 0.17; *P* = .20), both of which were substantially smaller than the functional RVI for MDD (Cohen *d* = 0.33-0.90).

### Functional RVI and Depression Symptoms

The functional RVI not only showed substantial effect sizes (Cohen *d* = 0.33-0.90) comparing MDD diagnosis vs controls but also had significant associations with quantitative depression symptom severity ratings across 3 samples, while structural RVI showed no significant correlation with any of the rating instruments (eResults in Supplement 1).

## Discussion

We examined the pattern of the ReHo deficits in individuals with MDD and report 3 novel findings: (1) the regional effect sizes for ReHo in participants with MDD vs controls were replicable across independent cohorts and were stronger than those for cortical thickness, (2) the regional pattern of lower ReHo signal agreed with the regional pattern of lower RCBF, and this finding was consistent regardless of the methodological approach to measure RCBF; and (3) the functional RVI for MDD that used ReHo deficit patterns showed stronger effect sizes than any structural RVI measurements and was significantly correlated with the severity of depression symptoms across samples. We interpret these findings as evidence for a reproducible pattern of regional hypoperfusion in MDD, suggested previously,^13,48,49^ and that this pattern can be captured from widely available resting functional MRI data.

The main novel finding is that the cortical pattern of reduced ReHo for MDD derived from the UKBB was significantly correlated with the cortical patterns of reduced ReHo and/or reduced RCBF in independent groups, ie, a non–treatment-seeking rural population sample in ACP (Figure 3E and F) and a patient sample with MDD seeking specialty treatment in ACI (Figure 3G), with remarkably similar correlation coefficients. The similarity in regional pattern for ReHo and RCBF, as well as the RVI analysis that intends to concisely capture the regional pattern, provide corroborative evidence that the regional ReHo deficits in MDD form a replicable pattern consistent with hypoperfusion in a regionally specific manner in MDD. Regional hypoperfusion in cingulate and frontal regions in MDD was first described over 3 decades ago.^13,48,49^ Hypoperfusion of limbic-frontal-temporal circuitry in MDD is associated with the severity of the depression symptoms and treatment resistance,^50^ and it is informative of the clinical state and treatment outcome.^13,49,51,52^ Significant regional case-control differences in ReHo values are also commonly reported in MDD and other psychiatric disorders.^21,53,54,55^ Prior studies presented these findings as deficits in local synchronization and/or focal neural connectivity without supportive evidence. The consistency in regional findings between ReHo and CBF provides a plausible physiological mechanism by linking patterns of lower regional ReHo values to patterns of lower regional CBF. This evidence suggests that ReHo-based functional cortical deficit pattern may represent the RCBF hypoperfusion pattern in MDD.

Structural neuroimaging findings in MDD have experienced heterogeneity and poor reproducibility.^2,56,57^ Here, we show that the lack of reproducibility of structural findings in MDD is the result of small effect sizes. The regional cortical deficits in MDD form a consistent and replicable pattern, but the small effect sizes make it suboptimal to serve as a biomarker. There was no significant overlap between cortical thickness and ReHo regional effect sizes in MDD. Previous findings on anatomic vs ReHo relationship in MDD have been mixed, with reports of overlapping^58^ and nonoverlapping^59^ abnormalities. However, these analyses were performed in modest sample sizes (N = 21-50), where small effect sizes in structural measures may have rendered the findings of association between functional and structural measures unreliable. Here, we used a statistically powerful sample to show that the MDD-related ReHo deficits were unrelated to MDD-related deficits in cortical thickness, suggesting hypoperfusion rather than cortical atrophy as the likely mechanism.

We compared effect sizes for functional and structural RVI for MDD. Structural RVI was only significantly elevated in the UKBB sample (Cohen *d* = 0.09). Functional RVI were significantly elevated in participants with MDD in the ACP sample using both CBF and ReHo data (Cohen *d* = 0.36 and Cohen *d* = 0.33). The highest elevation (Cohen *d* = 0.90) in functional RVI was observed in the CBF data from ACI sample composed of individuals actively seeking treatment for depression. Overall, these findings further advanced the RVI construct, showing that a functional alternative of the RVI can capture the effects of MDD on the cortex more robustly than structural RVI. The higher ReHo and CBF-derived RVI not only indexed the MDD diagnosis effects across samples but also captured the depression symptom severity effects, whereas the association between structural RVI and symptom severity did not reach statistical significance.

The RVI approach is not new or specific to ReHo. It was first applied in schizophrenia where higher RVI values for schizophrenia have been linked to treatment resistance, cognitive deficits, and family risks for the disease.^45,60,61,62^ The RVI approach has been proposed as a prospective tool for early detection of brain patterns shifting toward a particular condition as an early predictive biomarker.^62,63^ The novelty of this work was to show the applicability of RVI for functional MRI measures, here using ReHo, instead of structural brain deficits. It also presents an opportunity for future work to study the effects of intervention on improving RCBF for MDD and other illness conditions because unlike CBF imaging, ReHo values were calculated from widely and readily collected resting-state functional MRI data.

### Limitations

This study has some limitations. The study is limited to MDD, and we have not assessed disease specificity; future studies are needed to compare the illness specificity of these findings. Another limitation is that although the ReHo measurements share variance with CBF, this relationship is likely to be both complex and region specific. Our approach was limited by the choice of a coarse cortical atlas for measuring regional effect sizes. Our goal was to perform a 1:1 comparison with ENIGMA effect sizes, and this limited the choice of the atlas. More fine-grained, disorder-specific atlases can lead to improvements in RVI effect sizes.^64^ It is also unlikely that the findings of this study (UKBB and ACP sample datasets were collected using Siemens 3-T scanners and multiband sequences with similar acquisition parameters) will translate across all resting-state functional MRI protocols. Follow-up studies should evaluate if deviation from this protocol shifts the contrast between ReHo and RCBF and/or affects the MDD deficit pattern. Although the ReHo measurements share variance with CBF, this relationship is complex and region specific. In this study, we only evaluated 1:1 analyses of the association of MDD with ReHo; we did not account for more a complex RCBF and ReHo relationship where RCBF changes are associated with more distal ReHo variations and vice versa. The study was also limited by the choice of the datasets. The ReHo patterns were derived from a sample of middle-aged, White participants of UKBB sample. Rebuilding this pattern using a more diverse dataset will be beneficial for producing patterns that will be more reproducible in younger and more diverse cohorts.

## Conclusions

Results of this case-control study suggest that the RVI construct can be extended to functional domain and can provide a stronger brain pattern-based biomarker for MDD than structural RVI. The ReHo-based cortical deficit pattern observed in MDD is related to its regional perfusion deficit pattern. This suggests a novel mechanistic interpretation of this readily available resting-state functional MRI measure and urges reexamination and reinterpretations of previous ReHo findings across neuropsychiatric illnesses. ReHo as a resting-state functional MRI technique may open new doors to the study of brain perfusions in future etiological and interventional studies of neuropsychiatric conditions.

## Notes

Supplement 1.
eMethods.

eResults.
**eTable.** Effect Sizes for Participants With MDD vs Healthy Controls Were Calculated for Regional ReHo, Cortical Thickness, and SPECT rCBF Data
eReferences.

Supplement 2.
Data Sharing Statement.
