# Supplementary material for: Functional vs Structural Cortical Deficit Pattern Biomarkers for Major Depressive Disorder
Source: JAMA Psychiatry. 2025 Apr 2;82(6):582–90. doi: 10.1001/jamapsychiatry.2025.0192 (PMC11966481; doi:10.1001/jamapsychiatry.2025.0192)
Supplement: Supplement 1. — eMethods. eResults. eTable. Effect Sizes for Participants With MDD vs Healthy Controls Were Calculated for Regional ReHo, Cortical Thickness, and SPECT rCBF Data eReferences. [file jamapsychiatry-e250192-s001.pdf]

Supplementary Online Content

Kochunov P, Adhikari BM, Keator D, et al. Functional vs structural cortical deficit pattern biomarkers for major depressive disorder. *JAMA Psychiatry*. Published online April 2, 2025. doi:10.1001/jamapsychiatry.2025.0192

eMethods.

eResults.

eTable. Effect Sizes for Participants With MDD vs Healthy Controls Were Calculated for Regional ReHo, Cortical Thickness, and SPECT rCBF Data

eReferences

This supplementary material has been provided by the authors to give readers additional information about their work.

© 2025 Kochunov P et al. *JAMA Psychiatry*.

eMethods

UKBB sample

The UKBB sample was primarily used to calculate the MDD ReHo effect sizes. We also used the cortical thickness data to compare cortical thickness vs. ReHo effect sizes for MDD. The UKBB sample consisted of 18,898 participants (8,833/10,065 M/F, age: mean ± s.d.=63.2 ± 7.5 years), with resting state fMRI (rsfMRI), structural imaging and diagnostic information. We used the UKBB parser software (https://github.com/USC-IGC/ukbb_parser) to identify participants with recurrent MDD and non-psychiatric controls based on ICD codes, medication information, symptom severity, hospital records and self-reported diagnoses and other variables using previously published schema[1]. The recurrent MDD sample of *N*=2,220 (1,099/1,121 M/F, age: mean ± s.d.=63.2 ± 7.2 years) experienced between 2 and 8 (average=5.7 ±2.8) major depressive episodes in their lifetime[1]. N=2,590 (1094/1.496 M/F) control subjects (age: mean±s.d.: 62.9±7.2 years) were free of MDD and any other mental condition. Following the approach of a previous study [2], we quantified participants' depressive symptoms between 0 and 4 using two questions from the Patient Health Questionnaire (PHQ)-2 [3], one question was from the PHQ-9 [4] that measured fatigue ('felt tired or had little energy'). The last question measured psychomotor agitation ('felt tense, fidgety, or restless'). Participants rated their symptoms over the past 2 weeks from 'not at all' to 'nearly every day' (0–3). The sum of depressive symptoms was transformed to a four-point scale to correct for skewness[2] .

ENIGMA sample

The ENIGMA-MDD cortical thickness meta-analytic effect sizes were calculated from structural

© 2025 Kochunov P et al. *JAMA Psychiatry*.

MRI scans from *N*=2,148 MDD patients and *N*=7,957 healthy controls (age: mean ± s.d.=39.9 ±10.0 years) from 20 sites around the world. The effect sizes for cortical thickness are shown in **Table S1** and averaged for both hemispheres[5]. ENIGMA effect sizes were corrected for age, sex and their interaction.

ACP Sample

This sample was used for the primary hypotheses testing because it provided the cortical thickness, ReHo, and rCBF data that were collected within the same session. This family-based cohort from the ACP consisted of *N*=300 Amish participants (179/121 M/F, age: mean ± s.d.=37.5±16.3 years) with structural, rsfMRI and 3D-ASL data available (https://www.humanconnectome.org/study/amish-connectome-project). We limited this analysis to *N*=204 subjects, a sample that included *N*=68 (16/52 M/F, age: mean±s.d.=41.0±12.9 years) participants with a lifetime diagnosis of MDD and *N*=136 (63/73 M/F, age: mean±s.d.=41.0± 15.3 years) controls. Severity of depression symptoms were assessed using Beck Depression Inventory (BDI) [6] and the Maryland Trait and State Depression Scale (MTSD)[7]. BDI is composed of 21 questions to measure current severity of depression symptoms. MTSD assesses life-long trait depression severity and recent state depression severity, each consisted of 18 questions[7]. The total scores of each were used to measure trait depression and recent depression, respectively; more details are in http://www.mdbrain.org/MTSD_instructions_and_scale.pdf. We excluded *N*=96 participants with psychiatric and neurological disorders. Amish participants share a similar rural upbringing and lifestyle that includes the same level of basic school education (up to 8th grade), diet, and occupations, and virtually no illicit substance use, thus limiting the impact of potential confounding variables contributing to cerebral blood flow (CBF) variability present

© 2025 Kochunov P et al. *JAMA Psychiatry*.

in most populations. ACP exclusion criteria included major medical and neurological conditions that might affect gross brain structures - such as developmental disability, head trauma, seizure, stroke, or transient ischemic attack, and major body illnesses. All participants provided written informed consent on forms approved by the IRB of University of Maryland Baltimore.

ACI Sample

This sample provided SPECT measured CBF data that were collected to test for rCBF deficit patterns. The MDD participants were treatment-seeking patients under evaluation in ACI facilities. SPECT scans were acquired as part of patients' standard evaluation and prior to any treatments. We obtained the sample that included *N*=296 patients with recurrent MDD (*N*=183/113 M/F, age: mean±s.d.=46.1±17.2 years) and *N*=76 normal controls (34/42 M/F, age: mean ± s.d.=42.2± 17.2 years). Exclusion criteria included diagnosis of any mental illness other than MDD or any other major neuropsychiatric condition. All patients provided informed consent for their anonymous data to be used in future research, during their initial visit to the clinic (Integ Review Board #004-Amen Clinics Inc.). The severity of the depression symptoms upon admission was assessed using the total score of the PHQ-9 questionnaire[4].

Data Acquisition, Processing, and ReHo Analyses in UKBB and ACP

UKBB rsfMRI data were acquired on 3 T Siemens Skyra scanners with the standard Siemens 32-channel receiver head coil using the following parameters: TR=735 ms, TE=39 ms, spatial resolution of 2.4-mm isotropic voxels, matrix size=88×88 with 64 axial slices, number of volumes=490, flip angle=52°, and multi-band acceleration factor=8. A separate single-band reference image was acquired and used as the reference scan for head motion correction and

© 2025 Kochunov P et al. *JAMA Psychiatry*.

alignment to other modalities[8].

ACP participants underwent two runs of rsfMRI data acquisition, each approximately 6 minutes long. Oblique axial acquisitions alternated between phase encoding in the anterior-to-posterior (AP) and posterior-to-anterior (PA) directions within a single run. Separate single-band reference images, acquired for phase encoding in AP and PA directions, were used for spatial distortion correction. Each rsfMRI session was acquired using the following parameters: TR=780 ms, TE=34.4 ms, spatial resolution of 2-mm isotropic voxels, matrix size=104×104 with 72 axial slices, number of volumes=420, flip angle=52°, multi-band acceleration factor=8, and bandwidth=2,186 Hz/pixel. The two sessions led to a corrected dataset of 840 volumes.

The resting state analysis workflow developed by the ENIGMA consortium was used to process the rsfMRI data; processing steps have been described in full detail in prior publications[9,10]. The analysis workflow uses Marchenko-Pastur principal component analysis denoising[11] to improve signal-to noise ratio (SNR)/temporal SNR of the time series data. In this workflow, a transformation is computed registering the base volume to the ENIGMA EPI template, which is used as a common anatomical spatial reference frame for registration purposes. This step was followed by three-dimensional (3D) deconvolution of methodological covariates, and regression of the global signal[12]. Each functional volume was registered to the volume with the minimum outlier fraction for head motion correction, where each transformation was concatenated with the transformation to standard space, to avoid unnecessary interpolation. We removed the effects of the following nuisance variables by using them as covariates in the multiple linear regression analysis: the six (three rotations and three translations) motion parameters and their temporal derivatives, and time courses from the local white matter and cerebrospinal fluid from lateral

© 2025 Kochunov P et al. *JAMA Psychiatry*.

ventricles. Motion was estimated as the magnitude of displacement from one time point to the next including neighboring time points and outlier voxels fraction (> 0.1). Time points with excessive outlier voxels fraction and excessive motion (>0.2 mm) were censored and excluded from further statistical analysis. The AFNI-based minimum smoothing kernels of 4 mm (full width at half maximum) was included in the ENIGMA rsfMRI pipeline, but temporal filtering step was not included in the central preprocessing. Images were spatially normalized to the ENIGMA EPI template in MNI standard space for group analysis. The preprocessed data was then used for ReHo calculations.

ReHo was designed to investigate changes in regional temporal coherence in BOLD time-series by calculating voxelwise maps of Kendall's coefficients of concordance (KCC) [13]. The KCC score is calculated per voxel based on signals from neighboring voxels as: $W = \frac{\Sigma(R_i^2)-nR^2}{\frac{1}{12}K^2(n^3-n)}$. Here, W is the KCC among given voxels, ranging from 0 to 1; R_i is the sum rank of the ith time point; R $=((n+1)K)/2$ is the mean of the R_i's; K is the number of time series within a measured cluster (K is set to be 7, 19, or 27), and n is the number of ranks (n=number of volumes) [13]. K was set to be 27, which is appropriate for covering all directions in 3D space and to optimize the trade-off between mitigation of partial volume effects and generation of Gaussian random fields [14]. For each subject, the ReHo map was computed in three-dimensional volumetric space using the AFNI-command '*3dReHo*'. ReHo signals were extracted using cortical gray matter (GM) regions based on the Desikan–Killiany (DK) atlas. This atlas was used in the original ENIGMA cortical thickness data analysis. As our goal is to examine structural, ReHo and rCBF regional imaging data together, it is important to use the same atlas for all the imaging data.

© 2025 Kochunov P et al. *JAMA Psychiatry*.

Arterial-Spin Labeling Data Acquisition, Processing, and CBF Extraction in ACP

The ASL data were acquired using 3D pseudo-continuous ASL (pCASL) with background suppressed gradient and spin-echo sequence consisting of 13 pairs of labeled and control scans. The acquisition parameters were spatial resolution=2.5 mm×2.5 mm×2.5 mm, matrix size=96×96 with 58 axial slices, repetition time/echo time (TR/TE) =4,000/37 ms, flip angle=120°, field of view (FoV) read=220 mm, FoV phase=100%, post-label delay=1,700 ms, labeling duration=1,650 ms. Total scan time was approximately 10 minutes. A 3D T_1-weighted image was acquired for anatomical reference, as well as GM and white matter (WM) tissue segmentation, with the following parameters: TR=2,400 ms, TE=2.22 ms, inversion time=1,000 ms, flip angle=8°, matrix=300×320, slices per slab=208, 0.8 mm×0.8mm spatial resolution with slice thickness=0.80 mm. A volume of M_0 image was also acquired without background suppression to normalize the control-label difference for CBF quantification. The M_0 image was smoothed with a 5 mm Gaussian-kernel to suppress noise, per recent recommendations[15].

CBF data analysis was performed with the FSL software package; perfusion was estimated by using a standard single compartment ASL model; partial volume effects correction was performed with a spatially regularized method [16]. Spatial regularization, motion correction and partial volume corrections were performed in FSL v6.0.1. The high-resolution structural image provided partial volume estimates (PVE) for the different tissue types. The high-resolution PVE images obtained from a structural image were then converted to the ASL image space using a transformation matrix from the structural space to the ASL native image space. Partial volume corrected CBF maps were used to extract the voxel-wise CBF signals using volumetric Desikan-Killiany atlas that consisted of thirty-four cortical brain regions from each hemisphere. The right

© 2025 Kochunov P et al. *JAMA Psychiatry*.

and left measurements of the regions from the corresponding hemispheres were averaged.

SPECT Data Acquisition, Processing, and Analyses in ACI

SPECT scans were acquired using Picker (Philips) Prism XP 3000 triple-headed gamma camera (Picker Int. Inc., Ohio Nuclear Medicine Division, Bedford Hills, OH, USA) with low energy high resolution fan beam collimators. Subjects were scanned for approximately 30 minutes after injection. Data acquisition yielded 120 images per scan with each image separated by three degrees, spanning 360 degrees. A low pass filter was applied with a high cutoff and Chang attenuation correction performed. The final reconstructed image was 128×128×78 with the voxel size of 2.5×2.5×2.5 mm^3.

Statistical Analyses

Regional effect sizes for ReHo (in the UKBB and ACP), ASL rCBF (in ACP) and SPECT rCBF (in ACI) case-control differences were created using DK-atlas. ReHo effect sizes were quantified by comparing regional imaging data in the recurrent depression group and controls without neuropsychiatric diseases with the R packages 'effsize'[17] and 'psych'[18]. All calculations were adjusted for age and sex. SPECT effect sizes were created by comparing MDD patients and controls. In UKBB and ACP samples, we also calculated effect sizes for regional cortical thickness. These effect sizes were compared to the corresponding effect sizes reported by ENIGMA in the largest neuroimaging meta-analyses of MDD cases versus controls to date [5,19,20]. The reported significance of correlation coefficients among effect sizes was estimated using spin permutation test.

© 2025 Kochunov P et al. *JAMA Psychiatry*.

The regional effect sizes for ReHo and structural measurements were used to create the cortical ReHo and cortical thickness RVI scores based on previously established methods[21], using RVIpkg[22]. Briefly, RVI MDD scores were calculated by first regressing out the effects of age, sex, and intracranial volume from the imaging phenotypes for each individual and then inverse normalizing and standardizing the imaging measures to z-scores using the mean and standard deviation of the controls. The Pearson correlation coefficient was then calculated between the individual participants' z scores and corresponding effect sizes for metabolic versus control group differences previously created. All statistical analyses were performed in RStudio v4.1.1[23].

© 2025 Kochunov P et al. *JAMA Psychiatry*.

eResults.

Comparing MDD – Control Structural vs. ReHo Differences

There was no significant difference in regional cortical thickness between MDD cases and controls in the UKBB participants (d=-0.06 to 0.08, p>0.06, d means Cohen's d). Likewise, there were no significant cortical thickness MDD-related differences in the ACP cohort (d=-0.16 to 0.19, p>0.1) (**Table S1**). However, in this study, we show that the lack of reproducibility of structural findings in MDD is the result of small effect sizes rather than due to a lack of a consistent regional pattern. For example, while the UKBB participants showed no significant difference in regional cortical thickness after multiple comparison correction, the regional effect sizes pattern was still significantly correlated with that reported by the ENIGMA (r=0.64) (**Figure 2**). A similar trend was observed in ACP subjects where ENIGMA-MDD regional effect sizes in cortical thickness was correlated with these measures in the ACP sample (r=0.48). Therefore, despite the small effect sizes, it is possible to generate reproducible findings with structural imaging data in MDD when we compare the disease pattern across the entire brain.

In the UKBB cohort, the effect sizes of ReHo between MDD and controls ranged from d=-0.28±0.08 to -0.00±0.03 across the 33 regions, with the largest MDD effect sizes for ReHo were observed for the caudal cingulate, followed by superior and transverse temporal gyri (d=-0.39, -0.39 and -0.38, $p<10^{-39}$), only 2 of the 33 regions showed no significant MDD diagnosis effect: the entorhinal and frontal pole areas (d =-0.09 and -0.10), after correcting for multiple comparisons (**Table S1**).

Comparing MDD – Control ReHo vs. rCBF Differences

© 2025 Kochunov P et al. *JAMA Psychiatry*.

In ACI (**Figure 3D**), the largest ESs were observed in the superior and middle temporal gyrus (*d* =-0.66 and -0.64), followed by the cingulate gyrus (*d*=-0.58), while there were no significant rCBF differences in entorhinal cortex and frontal pole areas (*d* = 0.05 and 0.03, respectively).

Functional RVI and Depression Symptoms

In UKBB individuals with recurrent depression (*N*=2,220), the cortical ReHo-RVI for MDD was significantly correlated with the UKBB depression severity score (r=0.09, $p=2\times10^{-4}$), while this correlation with structural RVI was not significant (r=0.02, p=0.3). In ACP sample, ReHo-RVI was also significantly correlated with Beck Depression Inventory (BDI) total score (r=0.27, p=0.02) and Maryland Trait and State Depression scale (MTSD) trait and state scores (r=0.34, p=0.001 and r=0.27, p=0.02, respectively). RVI for MDD calculated from ASL rCBF data in ACP was also significantly correlated with MTSD trait score (r=0.24, p=0.04), although not with the BDI or MTSD state scores (r=0.15, p=0.2 and r=0.21, p=0.1). In comparison, the structural RVI-MDD again did not show significant correlation with BDI or MTSD trait or state scores (r=0.22, 0.13 and 0.08, all p>0.05). Finally, the correlation between RVI for MDD calculated from SPEC CBF data in ACI was significant correlated with the total PHQ-9 depression score (r=0.22, $p=3\times10^{-5}$). We noted that trait depression had more robust and consistent associations with ReHo- and CBF-based RVI compared to state depression. This RVI-MDD association with trait depression finding is consistent with earlier observations that trait depression, an assessment of longitudinally experienced depression severity over lifetime [7], was more strongly linked to genetic and stress factors for depression [24-26] than state or recent depression-based measures.

© 2025 Kochunov P et al. *JAMA Psychiatry*.

eTable. Effect Sizes for Participants With MDD vs Healthy Controls Were Calculated for Regional ReHo, Cortical Thickness, and SPECT rCBF Data. Cortical thickness data for ENIGMA were taken from Table S5 [5].

Regions	ReHo-UKBB	Cortical Thickness UKBB	ASL-ACP	ReHo-ACP	Cortical Thickness ACP	SPECT-ACI	Cortical Thickness ENIGMA
Bank of super temporal sulcus	$d = -0.37$, $p = 1.1 \times 10^{-36}$	$d = 0.02$, $p = 0.30$	$d = -0.20$, $p = 5.6 \times 10^{-3}$	$d = -0.15$, $p = 0.04$	$d = 0.01$, $p = 0.40$	$d = -0.27$, $p = 0.01$	$d = -0.07$
Caudal anterior cingulate	$d = -0.39$, $p = 1.1 \times 10^{-40}$	$d = 0.00$, $p = 0.40$	$d = -0.34$, $p = 1.6 \times 10^{-5}$	$d = -0.24$ $p = 7.6 \times 10^{-4}$	$d = 0.03$, $p = 0.40$	$d = -0.58$, $p = 1.0 \times 10^{-7}$	$d = -0.06$
Caudal middle frontal	$d = -0.19$, $p = 2.4 \times 10^{-10}$	$d = -0.03$, $p = 0.20$	$d = -0.12$, $p = 0.09$	$d = -0.12$, $p = 0.10$	$d = 0.16$, $p = 0.15$	$d = -0.29$, $p = 9.0 \times 10^{-3}$	$d = 0.00$
Cuneus	$d = -0.28$, $p = 8.2 \times 10^{-22}$	$d = 0.05$, $p = 0.10$	$d = -0.18$, $p = 0.02$	$d = -0.14$, $p = 0.05$	$d = 0.16$, $p = 0.15$	$d = -0.33$, $p = 2.0 \times 10^{-3}$	$d = 0.05$
Entorhinal	$d = -0.09$, $p = 2.9 \times 10^{-3}$	$d = -0.03$, $p = 0.30$	$d = -0.13$, $p = 0.07$	$d = -0.23$, $p = 1.2 \times 10^{-3}$	d=0.08, $p = 0.30$	$d = 0.03$, $p = 0.40$	$d = -0.05$
Frontal pole	$d = -0.10$, $p = 1.8 \times 10^{-3}$	$d = -0.01$, $p = 0.40$	$d = -0.18$, $p = 0.01$	$d = -0.22$, $p = 2.0 \times 10^{-3}$	d=0.20, $p = 0.09$	$d = 0.06$, $p = 0.30$	$d = -0.04$
Fusiform	$d = -0.32$, $p = 8.8 \times 10^{-28}$	$d = -0.04$, $p = 0.10$	$d = -0.05$, $p = 0.46$	$d = -0.09$, $p = 0.22$	$d = 0.06$, $p = 0.40$	$d = -0.46$, $p = 3.0 \times 10^{-5}$	$d = -0.12$
Inferior parietal	$d = -0.21$, $p = 7.2 \times 10^{-13}$	$d = 0.01$, $p = 0.40$	$d = -0.26$, p= 5.1×10^{-4}	$d = -0.16$, $p = 0.02$	$d = 0.18$, $p = 0.12$	$d = -0.45$, $p = 4.0 \times 10^{-5}$	$d = -0.05$
Inferior temporal	$d = -0.22$, $p = 7.0 \times 10^{-14}$	$d = -0.01$, $p = 0.40$	$d = -0.29$, $p = 1.4 \times 10^{-4}$	$d = -0.26$, $p = 2.6 \times 10^{-4}$	$d = 0.09$, $p = 0.30$	$d = -0.27$, $p = 0.02$	$d = -0.08$
Insula	$d = -0.37$, $p = 1.0 \times 10^{-36}$	$d = -0.01$, $p = 0.40$	d=-0.13, p=0.07	$d = -0.17$, $p = 0.01$	$d = 0.01$, $p = 0.40$	$d = -0.39$, $p = 4.0 \times 10^{-4}$	$d = -0.11$
Isthmus cingulate	$d = -0.31$, $p = 6.2 \times 10^{-26}$	$d = 0.00$, $p = 0.40$	$d = -0.20$, $p = 5.8 \times 10^{-3}$	$d = -0.05$, $p = 0.44$	d = -0.17, $p = 0.13$	$d = -0.41$, $p = 2.0 \times 10^{-4}$	$d = -0.09$
Lateral occipital	$d = -0.32$, $p = 2.7 \times 10^{-28}$	$d = 0.05$, $p = 0.01$	$d = -0.19$, $p = 9.5 \times 10^{-3}$	$d = -0.19$, $p = 7.3 \times 10^{-3}$	$d = 0.30$, $p = 0.02$	$d = -0.36$, $p = 9.0 \times 10^{-4}$	$d = -0.01$
Lateral orbito-frontal	$d = -0.24$, $p = 5.0 \times 10^{-16}$	$d = -0.01$, $p = 0.40$	$d = -0.15$, $p = 0.03$	$d = -0.22$, $p = 1.8 \times 10^{-3}$	$d = 0.10$, $p = 0.30$	$d = -0.46$, $p = 3.0 \times 10^{-5}$	$d = -0.08$
Lingual	$d = -0.37$, $p = 3.9 \times 10^{-37}$	$d = 0.03$, $p = 0.30$	$d = -0.14$, $p = 0.05$	$d = -0.11$, $p = 0.11$	d = -0.03, $p = 0.40$	$d = -0.57$, $p = 2.0 \times 10^{-7}$	d=-0.03
Medial orbito-frontal	$d = -0.23$, $p = 4.5 \times 10^{-15}$	$d = -0.04$, $p = 0.20$	$d = -0.21$, $p = 5.3 \times 10^{-3}$	$d = -0.21$, $p = 3.6 \times 10^{-3}$	d = -0.11, $p = 0.30$	$d = -0.58$, $p = 1.0 \times 10^{-7}$	$d = -0.13$

© 2025 Kochunov P et al. *JAMA Psychiatry*.

Middle temporal	$d = -0.34$, $p = 3.7 \times 10^{-30}$	$d = -0.04$, $p = 0.10$	$d = -0.12$, $p = 0.10$	$d = -0.15$, $p = 0.04$	$d = 0.09$, $p = 0.30$	$d = -0.64$, $p = 6.0 \times 10^{-9}$	$d = -0.09$
Paracentral	$d = -0.28$, $p = 8.5 \times 10^{-21}$	$d = -0.02$, $p = 0.30$	$d = -0.16$, $p = 0.03$	$d = -0.14$, $p = 0.06$	$d = 0.01$, $p = 0.40$	$d = -0.41$, $p = 2.0 \times 10^{-4}$	d=-0.005
Parahippocampal	$d = -0.34$, $p = 4.5 \times 10^{-30}$	$d = -0.01$, $p = 0.40$	$d = -0.24$, $p = 1.5 \times 10^{-3}$	$d = -0.21$, $p = 2.8 \times 10^{-3}$	$d = 0.00$, $p = 0.90$	$d = -0.28$, $p = 0.01$	$d = -0.07$
Pars opercularis	$d = -0.27$, $p = 1.2 \times 10^{-20}$	$d = -0.04$, $p = 0.20$	$d = -0.30$, $p = 9.9 \times 10^{-5}$	$d = -0.25$, $p = 4.1 \times 10^{-4}$	$d = 0.12$, $p = 0.22$	$d = -0.66$, $p = 2.0 \times 10^{-9}$	$d = -0.04$
Pars orbitalis	$d = -0.24$, $p = 9.0 \times 10^{-16}$	$d = -0.03$, $p = 0.20$	$d = -0.15$, $p = 0.04$	$d = -0.21$, $p = 3.8 \times 10^{-3}$	$d = 0.18$, $p = 0.12$	$d = -0.27$, $p = 0.02$	$d = -0.07$
Pars triangularis	$d = -0.25$, $p = 1.2 \times 10^{-17}$	$d = -0.01$, $p = 0.40$	$d = -0.15$, $p = 0.11$	$d = -0.23$, $p = 1.6 \times 10^{-3}$	$d = 0.15$, $p = 0.18$	$d = -0.59$, $p = 7.8 \times 10^{-8}$	$d = -0.04$
Pericalcarine	$d = -0.29$, $p = 1.9 \times 10^{-22}$	$d = 0.08$, $p = 7.3 \times 1$[illegible]	$d = -0.11$, $p = 6.7 \times 10^{-3}$	$d = -0.21$, $p = 3.87 \times 10^{-}$[illegible]	$d = 0.17$, $p = 0.14$	$d = -0.13$, $p = 0.20$	$d = 0.09$
Postcentral	$d = -0.37$, $p = 5.1 \times 10^{-37}$	$d = 0.01$, $p = 0.40$	$d = -0.20$, $p = 0.03$	$d = -0.17$, $p = 0.02$	$d = 0.13$, $p = 0.21$	$d = -0.32$, $p = 4.0 \times 10^{-3}$	$d = 0.03$
Posterior cingulate	$d = -0.32$, $p = 7.5 \times 10^{-28}$	$d = -0.01$, $p = 0.40$	$d = -0.15$, $p = 0.17$	$d = -0.11$, $p = 0.12$	$d = 0.04$, $p = 0.4$	$d = -0.19$, $p = 0.08$	$d = -0.10$
Precentral	$d = -0.31$, $p = 1.2 \times 10^{-26}$	$d = -0.02$, $p = 0.30$	$d = -0.10$, $p = 0.02$	$d = -0.13$, $p = 0.06$	$d = 0.14$, $p = 0.19$	$d = -0.26$, $p = 0.02$	$d = -0.02$
Precuneus	$d = -0.31$, $p = 3.3 \times 10^{-25}$	$d = -0.01$, $p = 0.40$	$d = -0.17$, $p = 0.23$	$d = -0.24$, $p = 7.8 \times 10^{-4}$	$d = 0.08$, $p = 0.32$	$d = -0.37$, $p = 7.8 \times 10^{-4}$	$d = -0.01$
Rostral anterior cingulate	$d = -0.26$, $p = 7.9 \times 10^{-19}$	$d = -0.03$, $p = 0.20$	$d = -0.09$, $p = 0.03$	$d = -0.09$, $p = 0.19$	$d = 0.06$, $p = 0.34$	$d = -0.05$, $p = 0.40$	$d = -0.11$
Rostral middle frontal	$d = -0.19$, $p = 9.6 \times 10^{-11}$	$d = -0.03$, $p = 0.30$	$d = -0.15$, $p = 0.10$	$d = -0.11$, $p = 0.11$	$d = 0.16$, $p = 0.15$	$d = -0.52$, $p = 2.5 \times 10^{-6}$	$d = -0.04$
Superior frontal	$d = -0.19$, $p = 5.9 \times 10^{-11}$	$d = -0.06$, $p = 0.040$	$d = -0.12$, $p = 0.03$	$d = -0.15$, $p = 0.04$	$d = 0.15$, $p = 0.18$	$d = -0.42$, $p = 1.4 \times 10^{-4}$	$d = -0.07$
Superior parietal	$d = -0.26$, $p = 2.2 \times 10^{-18}$	$d = 0.04$, $p = 0.20$	$d = -0.15$, $p = 0.04$	$d = -0.15$, $p = 0.03$	$d = 0.23$, $p = 0.06$	$d = -0.29$, $p = 7.4 \times 10^{-3}$	$d = 0.01$
Superior temporal	$d = -0.39$, $p = 3.9 \times 10^{-40}$	$d = 0.01$, $p = 0.40$	$d = -0.15$, $p = 2.8 \times 10^{-}$[illegible]	$d = -0.16$, $p = 0.02$	$d = 0.01$, $p = 0.40$	$d = -0.66$, $p = 1.7 \times 10^{-9}$	$d = -0.01$
Supramarginal	$d = -0.35$, $p = 4.6 \times 10^{-32}$	$d = -0.04$, $p = 0.20$	$d = -0.22$, $p = 0.016$	$d = -0.25$, $p = 5.6 \times 10^{-4}$	$d = 0.10$, $p = 0.30$	$d = -0.51$, $p = 2.8 \times 10^{-6}$	$d = -0.05$
Transverse temporal	$d = -0.38$, $p = 1.2 \times 10^{-38}$	$d = 0.05$, $p = 0.10$	$d = -0.18$, $p = 0.013$	$d = -0.19$, $p = 6.4 \times 10^{-3}$	$d = 0.08$, $p = 0.30$	$d = -0.36$, $p = 9.1 \times 10^{-4}$	$d = -0.04$

© 2025 Kochunov P et al. *JAMA Psychiatry*.

eReferences.

1. Smith DJ, Nicholl BI, Cullen B, et al. Prevalence and characteristics of probable major depression and bipolar disorder within UK biobank: cross-sectional study of 172,751 participants. *PLoS One*. 2013;8(11):e75362. doi:10.1371/journal.pone.0075362
2. Arnau-Soler A, Adams MJ, Clarke TK, et al. A validation of the diathesis-stress model for depression in Generation Scotland. *Transl Psychiatry*. Jan 18 2019;9(1):25. doi:10.1038/s41398-018-0356-7
3. Kroenke K, Spitzer RL, Williams JB. The Patient Health Questionnaire-2: validity of a two-item depression screener. *Medical care*. Nov 2003;41(11):1284-92. doi:10.1097/01.mlr.0000093487.78664.3c
4. Spitzer RL, Kroenke K, Williams JB. Validation and utility of a self-report version of PRIME-MD: the PHQ primary care study. Primary Care Evaluation of Mental Disorders. Patient Health Questionnaire. *Jama*. Nov 10 1999;282(18):1737-44. doi:10.1001/jama.282.18.1737
5. Schmaal L, Hibar DP, Samann PG, et al. Cortical abnormalities in adults and adolescents with major depression based on brain scans from 20 cohorts worldwide in the ENIGMA Major Depressive Disorder Working Group. *Mol Psychiatry*. Jun 2017;22(6):900-909. doi:10.1038/mp.2016.60
6. Beck AT, Ward CH, Mendelson M, Mock J, Erbaugh J. An inventory for measuring depression. *Arch Gen Psychiatry*. Jun 1961;4:561-71. doi:10.1001/archpsyc.1961.01710120031004
7. Chiappelli J, Nugent KL, Thangavelu K, Searcy K, Hong LE. Assessment of trait and state aspects of depression in schizophrenia. *Schizophr Bull*. Jan 2014;40(1):132-42. doi:10.1093/schbul/sbt069
8. Alfaro-Almagro F, Jenkinson M, Bangerter NK, et al. Image processing and Quality Control for the first 10,000 brain imaging datasets from UK Biobank. *NeuroImage*. Feb 1 2018;166:400-424. doi:10.1016/j.neuroimage.2017.10.034
9. Adhikari BM, Jahanshad N, Shukla DK, et al. Heritability estimates on resting state fMRI data using ENIGMA analysis pipeline. *Pac Symp Biocomput*. 2018;23:307-318.
10. Adhikari BM, Jahanshad N, Shukla D, et al. Comparison of heritability estimates on resting state fMRI connectivity phenotypes using the ENIGMA analysis pipeline. *Hum Brain Mapp*. 2018;39(12):4893-4902.
11. Veraart J, Novikov DS, Christiaens D, Ades-Aron B, Sijbers J, Fieremans E. Denoising of diffusion MRI using random matrix theory. *NeuroImage*. Aug 11 2016;142:394-406. doi:10.1016/j.neuroimage.2016.08.016
12. Adhikari BM, Jahanshad N, Shukla DK, et al. A resting state fMRI analysis pipeline for pooling inference across diverse cohorts: An ENIGMA rs-fMRI Protocol. *Brain Imaging Behav*. 2019;13(5):1453-1467. doi:*https://doi.org/10.1007/s11682-018-9941-x*
13. Zang Y, Jiang T, Lu Y, He Y, Tian L. Regional homogeneity approach to fMRI data analysis. *NeuroImage*. May 2004;22(1):394-400. doi:10.1016/j.neuroimage.2003.12.030
14. Jiang L, Zuo XN. Regional Homogeneity: A Multimodal, Multiscale Neuroimaging Marker of the Human Connectome. *Neuroscientist*. Oct 2016;22(5):486-505. doi:10.1177/1073858415595004
15. Alsop DC, Detre JA, Golay X, et al. Recommended Implementation of Arterial Spin Labeled Perfusion MRI for Clinical Applications: A consensus of the ISMRM Perfusion Study Group and the European Consortium for ASL in Dementia. *Magn Reson Med* 2015;73:102-116.
16. Chappell MA, Groves AR, MacIntosh BJ, Donahue MJ, Jezzard P, Woolrich MW. Partial volume correction of multiple inversion time arterial spin labeling MRI data. *Magn Reson Med* 2011;65:1173-1183.

© 2025 Kochunov P et al. *JAMA Psychiatry*.

17. *effsize: Efficient Effect Size Computation*. Version R package version 0.8.1. 2020 https://CRAN.R-project.org/package=effsize.
18. *psych: Procedures for Psychological, Psychometric, and Personality Research*. Version R package version 2.1.9. 2021. https://CRAN.R-project.org/package=psych.
19. Schmaal L, Veltman DJ, van Erp TG, et al. Subcortical brain alterations in major depressive disorder: findings from the ENIGMA Major Depressive Disorder working group. *Mol Psychiatry*. Jun 2016;21(6):806-12. doi:10.1038/mp.2015.69
20. van Velzen LS, Kelly S, Isaev D, et al. White matter disturbances in major depressive disorder: a coordinated analysis across 20 international cohorts in the ENIGMA MDD working group. *Mol Psychiatry*. Jul 2020;25(7):1511-1525. doi:10.1038/s41380-019-0477-2
21. Kochunov P, Fan F, Ryan MC, et al. Translating ENIGMA schizophrenia findings using the regional vulnerability index: Association with cognition, symptoms, and disease trajectory. *Hum Brain Mapp*. May 28 2020;doi:10.1002/hbm.25045
22. *RVIpkg*. Version R package version 0.2.8. 2021. https://CRAN.R-project.org/package=RVIpkg
23. RStudioTeam. RStudio: Integrated Development for R. 2020;
24. Humphries EM, Ahn K, Kember RL, et al. Genome-wide significant risk loci for mood disorders in the Old Order Amish founder population. *Mol Psychiatry*. Mar 7 2023;doi:10.1038/s41380-023-02014-1
25. Chiappelli J, Adhikari BM, Kvarta MD, et al. Depression, stress, and regional cerebral blood flow. *Journal of cerebral blood flow and metabolism : official journal of the International Society of Cerebral Blood Flow and Metabolism*. Jan 6 2023;271678x221148979. doi:10.1177/0271678x221148979
26. Kvarta MD, Bruce HA, Chiappelli J, et al. Multiple dimensions of stress vs. genetic effects on depression. *Transl Psychiatry*. Apr 29 2021;11(1):254. doi:10.1038/s41398-021-01369-9

© 2025 Kochunov P et al. *JAMA Psychiatry*.
