# Supplementary material for: Functional vs Structural Cortical Deficit Pattern Biomarkers for Major Depressive Disorder
Source: JAMA Psychiatry. 2025 Apr 2;82(6):582–90. doi: 10.1001/jamapsychiatry.2025.0192 (PMC11966481; doi:10.1001/jamapsychiatry.2025.0192)
Supplement: Supplement 2. — Data Sharing Statement. [file jamapsychiatry-e250192-s002.pdf]

Data Sharing Statement

Kochunov. Functional vs Structural Cortical Deficit Pattern Biomarkers for Major Depressive Disorder. *JAMA Psychiatry*. Published April 02, 2025. doi:10.1001/jamapsychiatry.2025.0192

Data
Data available: Yes
Data types: Other (please specify)
Additional Information: The UKBB and ENIGMA data are available from the corresponding studies. The ACP data is available via NIH-NDA.
How to access data: The UKBB and ENIGMA data are available from the corresponding studies. The ACP data will be made available via NDA permission.
When available: With publication

Supporting Documents
Document types: None

Additional Information
Who can access the data: ENIGMA data is freely available. UKBB and ACP/ACI data are available for researchers whose proposed use of the data has been approved by corresponding review board.
Types of analyses: The data will be made available for any research purpose.
Mechanisms of data availability: Data will be made available with a signed data access agreement.
